# A dielectric free near field phase transforming structure for wideband gain enhancement of antennas

**DOI:** 10.1038/s41598-021-93975-2

**Published:** 2021-07-28

**Authors:** Foez Ahmed, Muhammad U. Afzal, Touseef Hayat, Karu P. Esselle, Dushmantha N. Thalakotuna

**Affiliations:** 1 School of Electrical and Data Engineering, University of Technology Sydney (UTS), Sydney, NSW 2007 Australia; 2 School of Engineering, Macquarie University, Sydney, NSW 2109 Australia

**Keywords:** Engineering, Electrical and electronic engineering

## Abstract

The gain of some aperture antennas can be significantly increased by making the antenna near-field phase distribution more uniform, using a phase-transformation structure. A novel dielectric-free phase transforming structure (DF-PTS) is presented in this paper for this purpose, and its ability to correct the aperture phase distribution of a resonant cavity antenna (RCA) over a much wider bandwidth is demonstrated. As opposed to printed multilayered metasurfaces, all the cells in crucial locations of the DF-PTS have a phase response that tracks the phase error of the RCA over a large bandwidth, and in addition have wideband transmission characteristics, resulting in a wideband antenna system. The new DF-PTS, made of three thin metal sheets each containing modified-eight-arm-asterisk-shaped slots, is significantly stronger than the previous DF-PTS, which requires thin and long metal interconnects between metal patches. The third advantage of the new DF-PTS is, all phase transformation cells in it are highly transparent, each with a transmission magnitude greater than − 1 dB at the design frequency, ensuring excellent phase correction with minimal effect on aperture amplitude distribution. With the DF-PTS, RCA gain increases to 20.1 dBi, which is significantly greater than its 10.7 dBi gain without the DF-PTS. The measured 10-dB return loss bandwidth and the 3-dB gain bandwidth of the RCA with DF-PTS are 46% and 12%, respectively.

## Introduction

High-gain antennas with directive beams are essential to enhance the efficiency of wireless networks in long-distance communication systems including satellite communication, wireless backhaul networks, and other point-to-point microwave links. Parabolic reflector antennas^1–3^ are the most popular for high-gain applications, but due to their non-planar configuration, there has been a strong interest to find planar alternatives. In contrast, high gain (> 30 dB) large arrays of low-gain radiating elements^4–6^ are thin, planar and easily concealable but need a feeding network, which introduces additional complications and losses, particularly at millimetre wave frequencies. A reflectarray is a semi-planar solution where a planar array replaces the “dish”, yet it requires a horn or similar feed antenna at the focal distance^7–9^. Similarly, conventional transmitarrays also require focal-length separation between the feed antenna and the array that adds to the overall height of the antenna system^10–13^. This height was later reduced by folded reflect and transmit arrays^14–16^. As an example, in^14^, using the folded transmitarray, the spacing between the feed and transmit array was reduced from 16.2$\lambda _0$ to 4.75$\lambda _0$. However, the unit cell proposed in^14^ can only provide four discrete phase values (0, 90, 180, 270°) with a very large step of 90 degrees between them and also transform the polarization of the wave. Such a unit cell would not work in a compact (i.e. short, lower profile) near-field systems such as the one proposed here due to the continuous phase variation of the base antenna^17^. In the last decade, a great emphasis has been on metasurface-inspired devices and antennas^18–21^. Some antennas, such as modulated metasurface antennas, demonstrated high-gain with flat profiles^22–24^.

Extensive research on alternate compact high-gain antenna technologies led to the development of resonant cavity antennas (RCAs), which are also known as Fabry–Perot antennas, Electromagnetic Band-Gap (EBG) resonator antennas, Partial reflecting surface (PRS) antennas or 2D leaky-wave antennas^25–30^. The classical RCAs suffer from non-uniform phase distribution in the aperture field, which can be improved using a near-field phase-correcting structure (PCS), which is placed very close and parallel to the antenna aperture (at less than a wavelength spacing from the RCA). A previous printed multilayered phase-correcting structure has increased the gain of a classical RCA by 9 dB^17^.

The multilayered printed or all-dielectric phase-correcting structures^17,31–37^ are costly, due to the need for low-loss microwave laminates and specialized manufacturing that includes bonding multiple printed layers into a single metasurface. Moreover, the presence of dielectrics in the structure limits their potential applications in space (due to ionization), high-power microwave systems, and lightweight systems. An attempt to address these challenges for near-field phase transforming structure was made in^38^ where a fully metallic phase-correcting structure was designed without using any dielectric materials. The all-metal PCS is made of three thin metal sheets where each sheet has a different two-dimensional (2D) array of square conducting patches. This design draws its inspiration from the multilayered dielectric based printed metasurfaces, but with a notable difference that instead of dielectric laminates, the array of conducting patches are mechanically supported by a grid of thin metallic strips. The width of these metal strips in the grid is only 0.4 mm, which made metasurface fabrication complicated and significantly compromised the mechanical strength and stability of the metasurface when hundreds of conducting patches are required for a typical metasurface, for example, to correct near-field phase distribution of a high-gain antenna.

**Figure 1:**
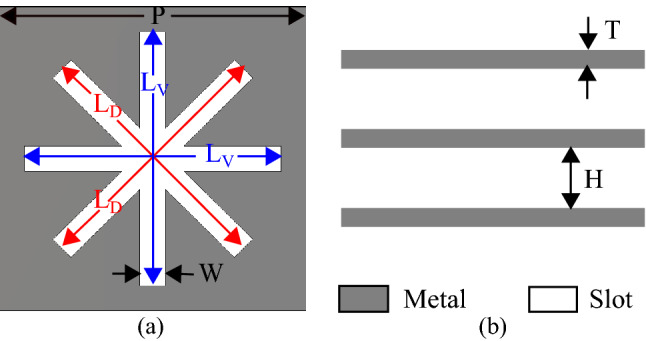
Configuration of the dielectric-free phase-shifting cell: (**a**) Top view; (**b**) Side view showing the three metal layers.

This paper addresses the challenge of mechanical stability and presents a novel dielectric-free phase transforming structure (DF-PTS). The DF-PTS is a 2D array of dielectric-free phase-shifting cells (DF-PSCs). The configuration of the new DF-PSC is shown in Fig. 1. Note that it has three slots, each having the shape of a Modified Eight-Arm Asterisk (MEAA), cut in three conducting (e.g. metal) sheets. Unlike in a standard asterisk, the lengths of the diagonal arms of this modified asterisk ($L_D$) are different from those of vertical and horizontal arms ($L_V$). Such DF-PSCs with different slot parameters are 2D-arrayed to form the new DF-PTS. The novelty of this work, compared to^38^, is that the DF-PTS proposed in this manuscript is based on slots that are cut in a continuous metal sheet, whereas the one in^38^ is based on metal patches. To keep all metal patches in place (in air), the patch-based approach requires narrow metal strips to connect all patches together. They are electromagnetically undesirable but essential to keep all the patches in place. Wide connecting strips would degrade performance, whereas narrow strips would compromise the mechanical stability of the phase transforming structure. The slot-based approach proposed here does not suffer from this limitation. Furthermore, narrower slots in the DF-PTS are fabrication friendly. They are easy to create with laser cutting, as opposed to the design in^38^, where removing large chunks of metal needs much longer exposure to the laser, which deforms the structure. Besides, a significant advantage is when the new DF-PTS is applied to correct the phase of a classical RCA, the measured 3-dB gain bandwidth of the antenna system after phase correction is significantly larger (69% improvement from 7.1% to 12.0%), compared to the antenna system with the previous DF-PTS with connected patches^38^. In fact, the measured 3-dB gain bandwidth with the new DF-PTS is significantly larger than the best bandwidth of 9.4% so far achieved using printed multilayered phase correction structure^32^ also. This paper compares antennas with the new DF-PTS and conventional printed metasurface (PM) and explains in detail why the new metasurfaces give significantly larger gain bandwidth.

Furthermore, the proposed DF-PTS does not alter the polarization of the electric field radiated by the base antenna. Due to the 90° (i.e. 4th Order) rotational symmetry of each DF-PSC, the new DF-PTS can be used to correct the near-field phase distribution of both linearly and circularly polarized antennas (including RHCP and LHCP and antennas with any other polarizations including dual LP and dual CP) without any design changes. Unlike^10^, the new DF-PTS is lightweight as it does not require any bulky guiding structure between metal sheets, which are simply suspended using spacers. It is to be mentioned here that, unlike the proposed structure, design in^10^ can only function with a circularly polarized wave (only RHCP or LHCP based on polarization alteration), not for the linearly polarized antennas.

## Method

### Dielectric-free phase shifting cell design

The fundamental building block of any near-field phase transforming structure is a phase-shifting cell (PSC). Unlike in printed multilayered metasurfaces where dielectric substrates provide mechanical support to multiple metal patch layers, a dielectric-free PSC (DF-PSC) made of metal sheets alone does not have a natural mechanical support. This additional challenge of mechanical stability becomes prominent as the antenna gain (and aperture area) increases. One solution is to interconnect metal patches using narrow metal strips, yet that approach provides only limited mechanical stability. To address this challenge, we propose a novel DF-PSC configuration that is slot-based as opposed to the patch-based approach in^38^. The narrow slots in the new DF-PSCs are created by removing a minimal amount of metal from the sheet, leaving plenty of metal to provide strong connections between cells.

The generic configuration of the new DF-PSC is shown in Fig. 1, which has slots in the shape of a modified eight-arm asterisk. All slots have a width denoted by *W*. The lateral length (*P*) of the cell is $\lambda _0/2$ ($=12$ mm), where $\lambda _0$ is the free-space wavelength at the example design frequency of 12.5 GHz. For the design example, the thickness (*T*) of metal sheets is assumed to be 0.5 mm (= $\lambda _0/48$). The complete DF-PSC has three identical layers, which are separated by a distance (*H*) of 6 mm (= $\lambda _0/4$) as shown in Fig. 1b.

**Figure 2:**
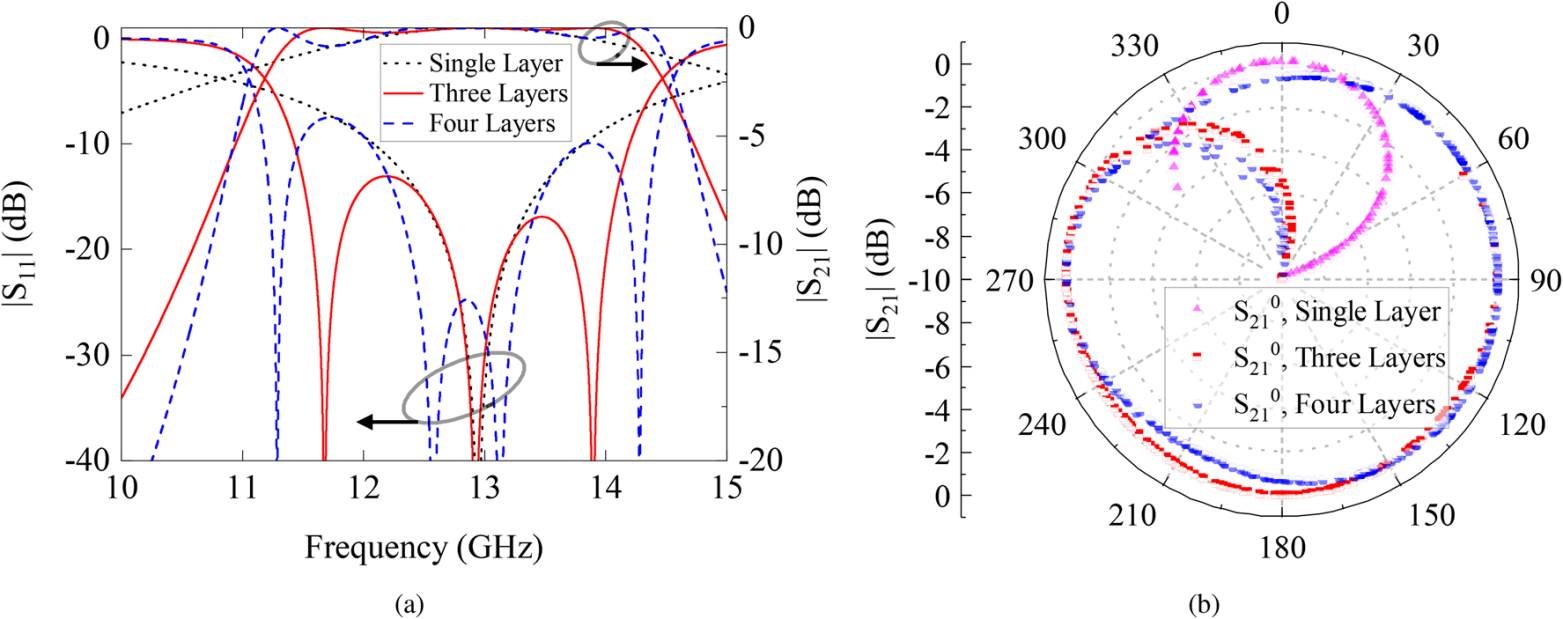
(**a**) Reflection and transmission characteristics of a DF-PSC with multiple identical metal layers, and (**b**) The transmission magnitude and phase of DF-PSCs on a polar plot.

The number of conducting layers in DF-PSC has a significant impact on its transmission and bandwidth characteristics. To explain this further, the DF-PSCs with a different number of metal layers were first simulated using the frequency-domain solver in CST Microwave Studio (MWS). These cells were analyzed with unit-cell boundary conditions and were excited with a plane wave propagating through the cells. The physical parameters $L_V$, $L_D$, and *W*, were set to 9 mm, 13.25 mm, and 1 mm, respectively. The transmission and reflection characteristics of the DF-PSCs with the different number of metal layers, compared in Fig. 2a, shows that each new layer generates an additional resonance in the frequency band and affects the transmission bandwidth of the DF-PSC. On the downside, additional layers add to the overall thickness of the structure. Therefore, a trade-off is needed amongst the bandwidth and thickness of the DF-PTSs.

DF-PSCs with one, three and four metal layers were considered. In each case, the slot width (*W*) was fixed to 1 mm and the lengths $L_D$ and $L_V$ were varied between 0.5 to 15 mm and 0.5 to 11 mm, respectively, in steps of 0.25 mm, to generate 2, 500 unique combinations of DF-PSCs. All these cells were simulated individually to determine the transmission characteristics of each (magnitude and phase). It is worth mentioning here that all the parameters of the DF-PSCs were chosen by considering the fabrication tolerances of local manufacturing facilities.

The transmission magnitude and phase of the 2, 500 cells were sorted, and the cells with poor performance were discarded. The transmission phase was normalized so that all phase values were between 0° and 360°. The normalized phase and transmission magnitude of the DF-PSCs are shown in a polar plot in Fig. 2b, for the cases of one, three and four metal sheets. A single-layer DF-PSC provides only a very limited 90° phase-shift range^39^ with high transmission magnitude larger than − 1 dB. The three-layer cells cover the phase-shift range between 60° and 320° while maintaining a high transmission magnitude greater than − 1 dB. The phase-shift range can be extended further by adding an identical fourth layer to the DF-PSC. The four-layer cells can provide the complete phase shift range from 0° and 360° with high transmission magnitude (see the blue dotted curve in Fig. 2b). However, as discussed previously, this comes at the expense of increased thickness, cost and weight. Hence, as a compromise, a three-layer DF-PSC was selected to develop the DF-PTS discussed in the following subsection. In simulations, the DF-PSCs were excited by linearly polarized plane waves. However, due to their 90° rotational symmetry, they are equally suitable for circularly polarized antenna systems.

### Design example

For an example, a DF-PTS was designed for the wideband gain enhancement of a resonant cavity antenna (RCA). As our intention was to find a wideband, low-cost, lightweight alternative to conventional printed multilayered near-field phase correction metasurfaces, to make a fair comparison, we also designed a conventional printed metasurface (PM) for this RCA. The antenna configuration, two structure designs and their performance comparison are presented in this subsection in detail.

#### Antenna configuration

**Figure 3:**
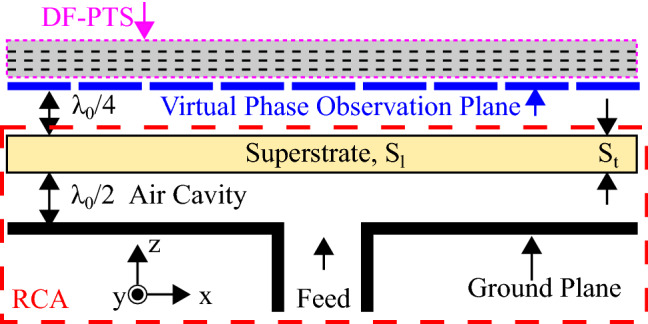
Cross section of the proposed antenna system where DF-PTS is placed in the virtual plane defined parallel to the RCA in the near-field region.

A detail cross-sectional view of the proposed antenna system is shown in Fig. 3. A typical RCA comprises a primary feed supported through a ground plane and a superstrate or a partially reflective surface (PRS). The PRS and the ground plane form a cavity that resonates at the operating frequency. Most RCAs have air-filled cavities, and the PRS and ground plane are separated by a distance that is approximately half the free-space wavelength at the operating frequency. In most cases (but not necessarily always) such a spacing ensures that the cavity resonates at the operating frequency^26^.

In a classic simple RCA configuration that is considered here and in many previous phase correction articles^17,31–35^ , the PRS is made of an unprinted all-dielectric superstrate (Rogers TMM4). The superstrate has a square shape with length ($S_l$) of $6\lambda _0$ ($=144$ mm), where $\lambda _0$ is the free-space wavelength at the design frequency of 12.5 GHz and thickness ($S_t$) of 3.18 mm. The superstrate and the ground plane are separated by $\lambda _0/2$ and form an air-filled cavity. The cavity is excited through a WR-75 waveguide-to-coaxial adapter, which is fixed to a 12 mm$\times 8$ mm feeding slot at the center of the ground plane. The superstrate is made of a relatively low-permittivity dielectric material with a dielectric constant ($\epsilon _r$) of 4.7 and thus has a low reflection magnitude, which leads to a non-uniform phase distribution in the electric field both inside and outside of the cavity^17,31^. The severity of non-uniformity in the aperture near-field phase distribution can be probed using virtual E-field probes in a full-wave simulation of the RCA model. The virtual plane on which the antenna near field is probed is defined at a quarter of free-space wavelength spacing from the top of the superstrate, as shown in Fig. 3. This choice is not arbitrary; this is the location where one intends to place the DF-PTS to correct phase non-uniformity of the RCA. Next, this plane is discretized into a 2D grid of equal-sized and uniformly distributed squares, and the phase is probed at the center of each square to create the phase map of the near-field distribution. These aspects of metasurfaces designs have been discussed at length in previous publications^17,31^ and are not repeated here for brevity.

It is worth emphasizing here that most of the antennas, such as microstrip-patch antennas and conical horn antennas, have a non-uniform aperture phase distribution. Hence, any of those can be used to prove the concept. However, the RCA is used as a base antenna here to make a fair performance comparison with previously reported phase-correcting structures that are mostly tested with a particular type of a simple classical RCA. Although we have used dielectric-superstate based RCA as a base antenna to demonstrate the DF-PTS, in actual high power application, an all-metal RCA design reported in^27^, which has a metal superstrate, can be used instead. Since the DF-PTS is placed very close to the RCA in its near-field region, it must be highly transmitting (i.e. every cell with transmission > − 1 dB) so that the DF-PTS does not affect the performance of the antenna system due to undesirable reflections.

#### Dielectric-free phase transforming structure (DF-PTS) design

**Figure 4:**
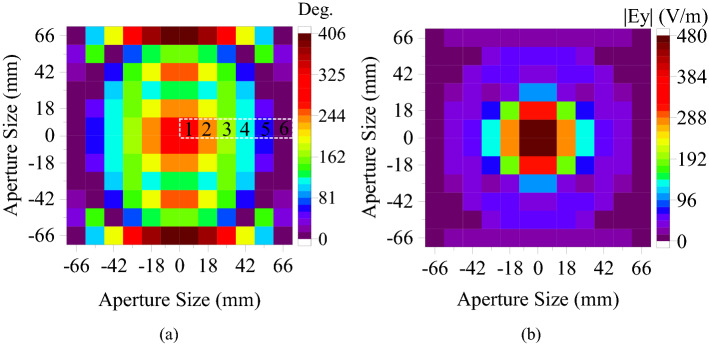
(**a**) The phase and (**b**) magnitude of the dominant electric field component $(E_y)$ on the aperture of the RCA.

The DF-PTS was designed following the general design strategy reported in^17^. First, the virtual phase observation plane (see Fig. 3) was divided into a 2D grid of 144 ($12\times 12$) equal-sized squares, where the size of each square is the same as that of the DF-PSC. The RCA model was simulated using CST MWS. Its dominant electric field component ($E_y$) of the near field was probed at the center of each square in the 2D grid. The magnitude and phase distributions of $E_y$ on RCA aperture are illustrated in Fig. 4. As observed in previous publications, the phase distribution of the RCAs is rotationally symmetric around the center of the aperture; hence, the whole structure was designed using the phase along one half of a linear axis.

The phase data of these six squares along the x-axis, which are labeled in Fig. 4a, was used to design the DF-PTS. The phase was normalized first. For the explanation of the design process, this probed phase is referred to as the input phase and denoted by $\theta _{IP}(x, y)$. The normalized phase values at the center of each square are given in Table 1. They were used to approximate the aperture near-field phase distribution of the RCA on the 2D plane by rotating the phase around the center of the aperture, as explained in^17,31^. However, the DF-PTS was designed by individually placing in each square of the grid an appropriate DF-PSC with the correct phase shift such that the non-uniform phase $\theta _{IP}(x, y)$ at the input of the DF-PTS is transformed into a nearly constant (uniform) phase at the output of the DF-PTS. The required ideal phase shift, $\Delta \theta (x, y)$, for each DF-PSC of the DF-PTS is given by

1 $$\begin{aligned} \Delta \theta (x, y) = \theta _{out}-\theta _{IP}(x, y) \end{aligned}$$

where $\theta _{out}$ is the desired constant phase at the output of the DF-PTS and (*x*, *y*) represents the center of the square in the 2D grid. The output phase is set to 376° to ensure that there exists a cell with a transmission phase close to the ideal phase $\Delta \theta (x, y)$ in each cell of the 2D grid.

The required $\Delta \theta (x, y)$ values for the DF-PSCs to be placed at the six squares highlighted in Fig. 4a are listed in Table 1. As an example, the probed phase at $x=18$ mm is 237°, and thus the DF-PSC needs an ideal phase shift of 139° (= 376°-237°). Then the data for three-layered DF-PSCs shown in Fig. 2b were used to select the best DF-PSC such that each has the highest transmission magnitude and an actual phase shift that is closest to the ideal phase shift required. Therefore, a DF-PSC with slot dimensions of $L_D=14.50$ mm and $L_V=3.00$ mm was selected. Note that the selected “best” cell has an excellent transmission magnitude of $-0.18$ dB and an actual phase shift of 136° (last column). There is a phase shift error of 3° in this cell, which is insignificant in the near-field phase transformation. The slot dimensions for the rest of the DF-PSCs are listed in Table 1.

**Table 1:** Center position, dimensions and transmission coefficient of each cell.

Location of the cell center *x* (mm)	Normalized phase $\theta _{IP}(x, y)$ (°)	Ideal phase shift $\Delta \theta (x, y)$ (°)	Cell slot dimensions and cell transmission coefficient			
			$L_D$ (mm)	$L_V$ (mm)	$|S_{21}|$ (dB)	Actual phase (°)
6	294	82	15.00	2.00	− 0.01	82
18	237	139	14.50	3.00	− 0.18	136
30	169	207	13.25	9.00	− 0.13	205
42	109	267	12.50	7.00	− 0.07	265
54	59	317	12.00	7.57	− 0.83	316
66	0	16	15.00	0.50	− 0.27	60

**Figure 5:**
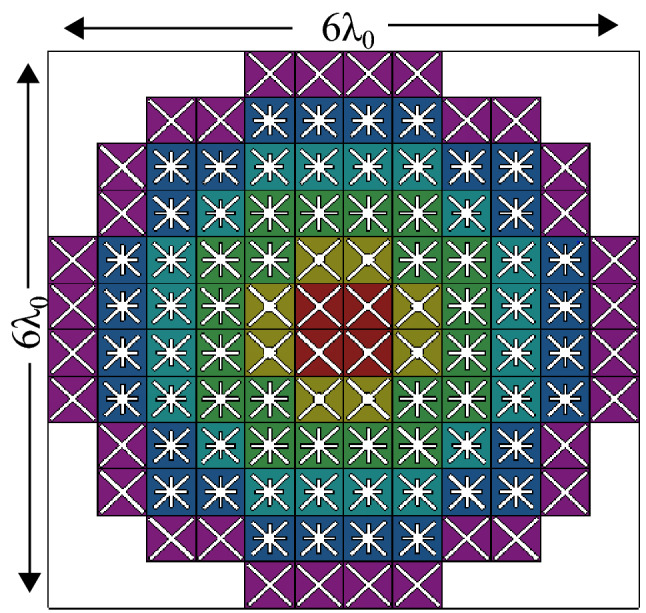
The layout of the dielectric-free phase-shifting cells in the DF-PTS.

It is worth mentioning that all DF-PSCs used in the DF-PTS design have a transmission magnitude greater than − 0.83 dB. The maximum difference between the ideal phase shift and the actual phase shift of the selected DF-PSCs is 3° except for the last cell, which has a difference of 44°. Since the last cell is located at the outermost edge of the aperture, it has a much lower near-field amplitude than the other cells, as shown in Fig. 4b. In the near-field phase transformation, phase errors in such low-amplitude cells can be accommodated without significant degradations of overall phase correction performance. Should such an error be in a high-amplitude cell, a noticeable performance degradation could result. If that happens, it can be prevented by selecting a DF-PSC with four layers, which can provide any phase shift with high transmission, as shown in Fig. 2b. However, the selected six DF-PSCs are arranged with approximate rotational symmetry, similar to that of the aperture phase, to form the 2D layout of the DF-PTS. The top layer of the DF-PTS model overlaid on phase-shift values is shown in Fig. 5. The RCA with DF-PTS was simulated using the time-domain solver in CST MW Studio. The cavity height and the spacing between the RCA and the DF-PTS were re-tuned to 13 mm and 7 mm, respectively. The height was tuned to mitigate the DF-PTS loading effects and to achieve maximum directivity/gain at the operating frequency. The DF-PTS can also be designed at a spacing less than $\lambda _0/4$ from the RCA. However, if the distance between the base antenna and the structure is less than $\lambda _0/4$, the coupling effect deteriorates overall antenna performance.

#### Printed metasurface (PM) design

**Figure 6:**
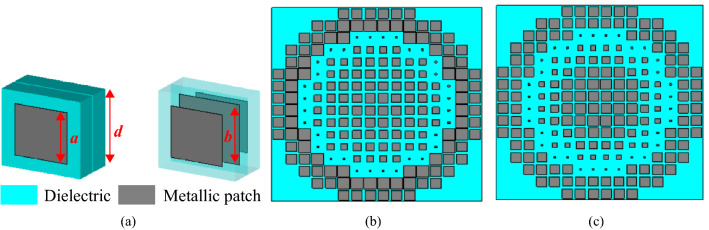
(**a**) Perspective view of the printed unit-cell configuration, and Layouts of the printed metasurface: (**b**) patches on the top layer (bottom layer is identical); (**c**) patches on the middle layer.

To compare with the new DF-PTS, we also designed a conventional printed metasurface (PM) operating at 12.5 GHz following the method that is well described in^17^. The configuration of the printed phase-shifting cell (P-PSC) used in PM is shown in Fig. 6a. It comprises two dielectric layers and three metal layers printed on the dielectrics. Each metal layer has a square patch. In a given cell, the lengths of the patches in the first and third layers are identical, which are represented by *a*. The length of the square patch in the middle layer is different and represented by *b*. The length *d* of the P-PSC is fixed to 9 mm, which is $0.375\lambda _0$ and is similar to that in^17^. The P-PSC was simulated with unit-cell boundary conditions, and the two parameters were varied from 0.5 to 8.75 mm. The PM was designed following the same method explained previously for the DF-PTS design. However, unlike six DF-PSCs, eight P-PSCs were required to cover one half of the RCA aperture. These eight cells can be precisely defined by dividing the RCA aperture grid symmetrically into 256 equal-sized squares. RCA field phase was probed at the center of eight squares along the x-axis, which was then used to calculate the required phase shift as before. Eight highly transmitting P-PSCs were picked according to the required phase shift. The PM was then formed using those selected eight printed PSCs. The layouts of the patches on the top and middle metal layers of the PM are shown in Fig. 6b,c. The RCA with PM was simulated using the time-domain solver of CST MWS. The RCA cavity height and the spacing between RCA and PM were tuned to 12 mm ($=\lambda _0/2$) and 6 mm ($=\lambda _0/4$), respectively, to achieve maximum directivity at the operating frequency.

#### Comparison of two phase transforming structure types

As mentioned previously, the new DF-PTS was proposed as an alternative to PMs. The performances of the two types of structures (DF-PTS and PM), when applied to phase correction of the RCA, are assessed and compared here, based on the results predicted through full-wave simulations.

**Figure 7:**
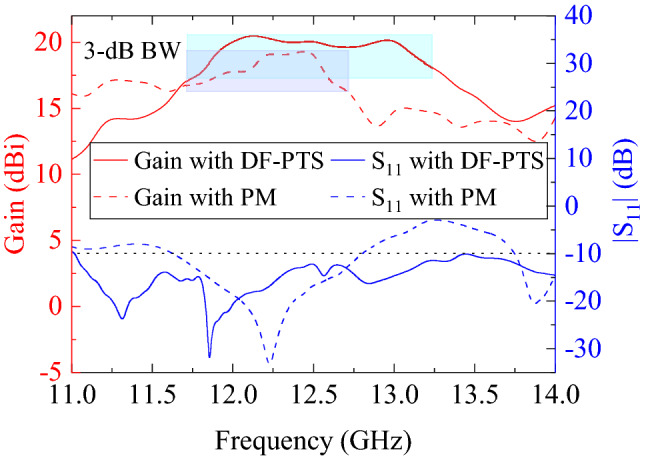
Comparison of input reflection magnitudes and peak gains of RCAs that are phase-corrected using DF-PTS and PM.

First of all, both structures have increased the gain of the RCA significantly, thanks to their near-field phase correction. The peak gain of the RCA without any phase correction is only 10.1 dBi at the design frequency of 12.5 GHz. With the DF-PTS it increases to 20.1 dBi and with PM it reaches fairly close to 19.5 dBi. Yet, there are significant differences in performance between the two approaches. The impedance matching and broadside gain of the RCAs that are phase-corrected using the DF-PTS and PM are plotted in Fig. 7. The key performance parameters of both antenna systems are listed in Table 2. The $-10$ dB impedance matching bandwidth of the RCA with DF-PTS is 11.0–17.5 GHz, which is significantly larger than the 11.63–12.8 GHz bandwidth of the RCA with PM. The 3-dB gain bandwidth of the RCA with DF-PTS is 11.7–13.2 GHz, which is 46% greater than the percentage bandwidth of the RCA with PM. It is also important to note that, with DF-PTS, the peak gain of the antenna system is more stable with frequency variations while it fluctuates a lot with PM.

**Table 2:** Performance Comparison of the RCA with PM and DF-PTS.

Parameters	RCA with PM	RCA with DF-PTS
Impedance BW (GHz)	1.2	6.5
Impedance BW (%)	9.5	46.0
3-dB gain BW (GHz)	1.0	1.5
3-dB gain BW (%)	8.2	12.0
Maximum peak gain (dBi)	19.5	20.6
Maximum peak directivity (dBi)	19.7	20.7

The gain-bandwidth with DF-PTS is the most striking difference. It is much larger than the gain-bandwidth of the same RCA when a PM of the same area is used for phase correction. To investigate this remarkable difference between the two types phase transforming structures, let us analyze further their constituent cells in a large frequency band. Generally, in an antenna system with a base antenna (i.e. the RCA here) and a phase correction structure, the two parameters that determine the system bandwidth are transmission bandwidths of the constituent cells and their phase response relative to the phase error response of the base antenna. To investigate the superiority of DF-PSCs, we simulated each of the six individual cells used in the DF-PTS in the frequency band from 11.7 GHz to 13.2 GHz, i.e. the 3-dB gain bandwidth of the final system.

**Figure 8:**
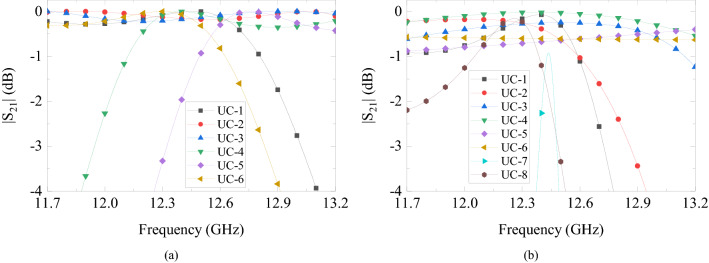
Transmission magnitudes of (**a**) DF-PSCs selected to design the DF-PTS, and (**b**) P-PSCs selected to design the PM.

The magnitudes of the transmission coefficient for all six cells are given in Fig. 8a. Two out of six cells have a transmission coefficient magnitude greater than − 3 dB throughout the frequency band between 11.7 GHz and 13.2 GHz. Two cells have a magnitude more than − 3 dB for 87% of the frequency band, while the two outermost cells have a higher than − 3 dB magnitude in 60% of the frequency band. To compare, results of a similar analysis for the eight cells used in the PM (see Fig. 6b,c) are shown in Fig. 8b. It can be seen that only half of these printed cells have transmission magnitudes greater than $-3$ dB throughout the band while the rest of the cells have a fractional $-3$ dB bandwidth, with one cell being an extremely narrow band. The large transmission bandwidth of the DF-PSCs can be attributed to the multiple resonances of the DF-PSC.

**Figure 9:**
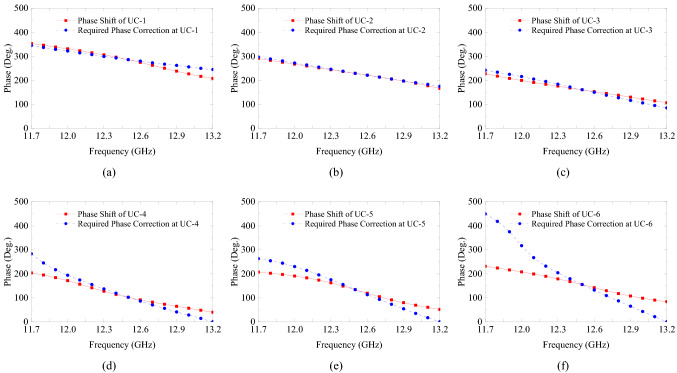
Phase shift of each DF-PSC and the phase correction required in the RCA aperture at each DF-PSC location versus frequency. (UC-1 is at the center of the antenna aperture and UC-6 is the outermost).

**Figure 10:**
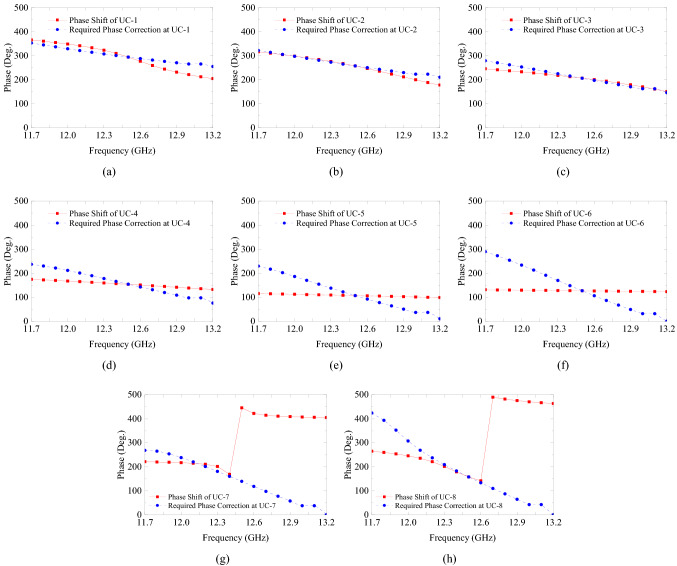
Phase shift of each P-PSC and the phase correction required in the RCA aperture at each P-PSC location versus frequency. (UC-1 is at the center of the antenna aperture and UC-8 is the outermost).

Next let us compare the phase responses of all the unit cells selected previously for the DF-PTS and the PM, Figs. 9 and 10, respectively. In order to have wideband phase correction performance, each phase correcting cell of the structure must have the ability to track the phase error of the base antenna at the cell location over a large frequency range. Therefore, these figures also show the phase correction required in the RCA aperture at each cell location versus frequency. This is the opposite of the phase error at this location in the dominant RCA aperture field $E_y$, and it is expected to be corrected by the phase transformation cell. For example, the phase of the center cell, UC-1 in DF-PTS, is plotted with the RCA phase correction required at (*x*, *y*) = (6 mm, 6 mm). Figure 9 indicates that all the DF-PSCs except the outermost cell (where field amplitude is the lowest) closely follow the phase error of the RCA with frequency, leading to the wideband phase correction we observed previously. On the contrary, the phase responses of most of the P-PSCs do not follow the phase error variation of RCA with frequency, as demonstrated in Fig. 10. Thus, when compared with a PM, phase correction with a DF-PTS leads to much larger gain and directivity bandwidths as a result of wideband phase correction and high-transmission magnitude bandwidth of nearly all cells.

## Experimental results

**Figure 11:**
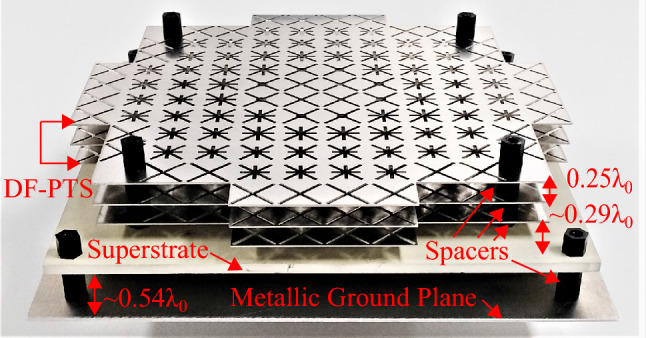
Photograph of the assembled prototype with the DF-PTS and the RCA.

Prototypes of the DF-PTS and RCA were developed and measured using Agilent PAN-X N5242*A* vector network analyzer and in an anechoic chamber to validate the concepts. The assembled antenna system is shown in Fig. 11. The three layers of the DF-PTS were manufactured using high-precision laser cutting technology. A sheet of Stainless Steel with a thickness of 0.5 mm ($=\lambda _0/48$) ± 3% was used to fabricate the layers of the DF-PTS. The fabrication tolerance of the laser cutting machine was about ± 20 $\mu$m. Three identical layers so fabricated through laser cutting were assembled together, using four spacers inserted through pre-cut holes near the four corners of each fabricated layer, to form the DF-PTS. The DF-PTS was fixed above the superstrate using four plastic spacers.

**Figure 12:**
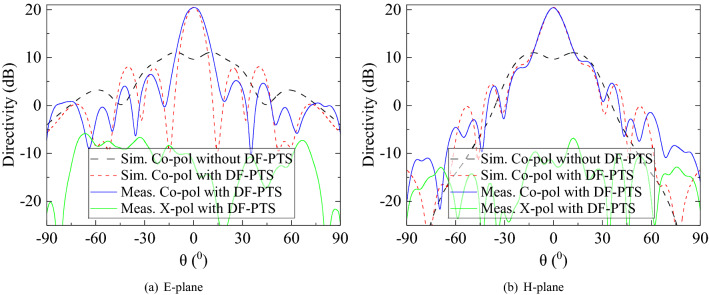
Measured and predicted far-field pattern cuts of the RCA with and without the DF-PTS, at 12.5 GHz.

**Figure 13:**
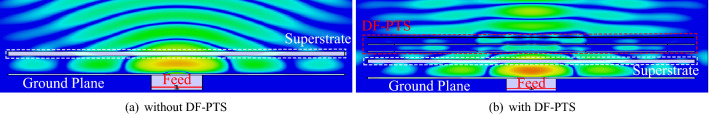
Snapshot of the near field distribution (dominant $E_y$ field component) in a cross-section of the antenna.

The far-field patterns were measured in an NSI spherical near-field antenna range. At the operating frequency of 12.5 GHz, the far-field pattern cuts of the RCA on two principal planes, with and without the DF-PTS, are compared in Fig. 12. The measured and predicted patterns are in good agreement with minor discrepancies. The peak gain of the RCA with the DF-PTS is 19.3 dBi, and the beam peak is in the broadside direction. The antenna system has excellent cross polar rejection in the direction of the beam peak, where the cross-polar component is 30 dB below the co-polar component. The measured sidelobe levels (SLLs) are greater than 15 dB and 16 dB in E-plane and H-planes, respectively. The antenna directivity increases by 10.1 dB at the design frequency when the DF-PTS is introduced to the RCA (see Fig. 12). This is due to the improvement in the uniformity of the antenna aperture phase distribution. This is confirmed in Fig. 13, which compares the near electric field phase of the RCA on the xz-plane with and without the DF-PTS. With DF-PTS, the phase fronts of the antenna are nearly planar, indicating strong uniformity in the phase distribution. It is to be emphasized here that cells designed with unit-cell boundary conditions when used in DF-PTS, placed in the near-field region of RCA, demonstrated anticipated phase correction. It is because the phase variation within the area equivalent to the size of the unit cell even within the near-field region is less than 45°.

**Figure 14:**
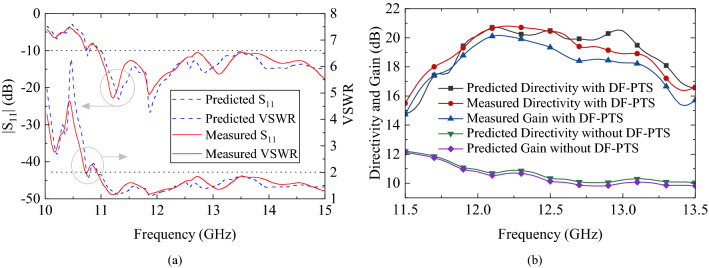
Measured and predicted (**a**) input impedance matching of the RCA with DF-PTS, and (**b**) peak directivity and peak gain of the RCA with and without DF-PTS.

**Figure 15:**
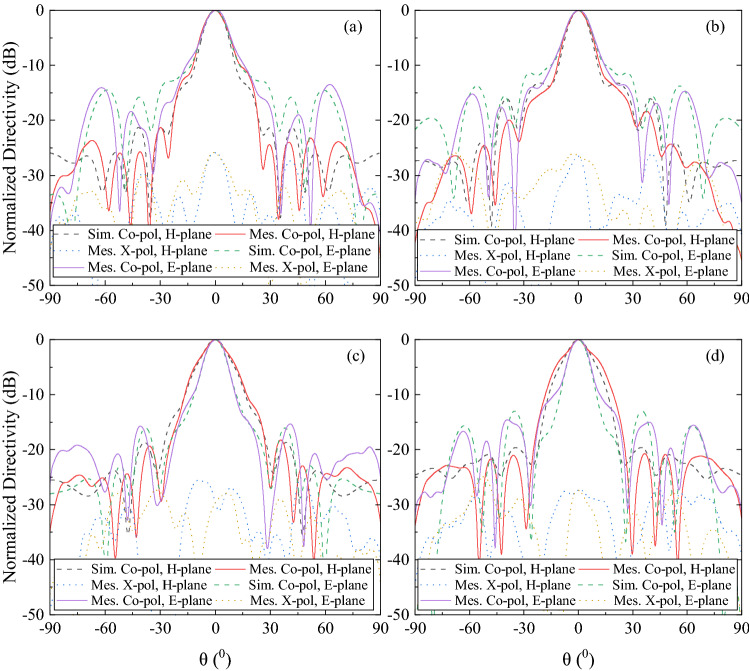
Measured and predicted far-field patterns of the RCA with DF-PTS at four frequencies. (**a**) 12.1 GHz, (**b**) 12.3 GHz, (**c**) 12.7 GHz and (**d**) 12.9 GHz.

Although the DF-PTS was designed at a single operating frequency, its performance was measured over a broad frequency range. The voltage standing wave ratio (VSWR) and $|S_{11}|$ of the RCA with DF-PTS are plotted in Fig. 14a. The measured 10-dB return loss bandwidth is approximately 46% (from 11.0 GHz to 17.5 GHz), which is in good agreement with what was predicted from a simulation. The measured and predicted peak directivity and peak gain of the RCA with and without the DF-PTS over a large frequency range are shown in Fig. 14b. The measured maximum gain of 20.1 dBi is noted at 12.2 GHz. At this frequency, the aperture efficiency of the RCA with DF-PTS is 24.0%. This is a dramatic increase compared to the 2.7% aperture efficiency of the RCA without the DF-PTS. The measured 3-dB directivity bandwidth of the RCA with DF-PTS is 11.7–13.2 GHz, which is 12.0% of the center operating frequency. The measured gain bandwidth of RCA with DF-PTS is 11.7–13.2 GHz or 12%, which is a dramatic 1.7-fold improvement over the fractional bandwidth achieved with the DF-PTS in^38^. The antenna system has a stable radiation pattern within the 3-dB frequency bandwidth. The normalized pattern cuts in E- and H-planes at four different frequencies within the band are shown in Fig. 15. For comparison, predicted patterns are also included in the plots, which indicate excellent agreement between the measured and predicted patterns. The sidelobes throughout the band are at least 15 dB below the main lobes, and the measured cross-polar levels in the direction of the main beam are at least 26 dB below the co-polar level.

## Discussion

The performance enhancement of the RCA achieved with the DF-PTS are quantitatively compared with published results in Table 3. Some popular figures-of-merit for comparison are peak gain, directivity and 3-dB gain and directivity bandwidths. In addition, a few other relevant figures-of-merit are also compared in Table 3. Since in most high-gain antennas, the peak directivity/gain and bandwidth are inversely related, directivity-bandwidth product (DBP) or gain-bandwidth product (GBP) are relevant indications of overall performance. Yet, both of them increase with antenna electrical area in general and do not represent area effectiveness. To bring aperture efficiency into the picture, DBP per area (DBP/A) and GBP per area (GBP/A) have been defined in the past^40^. These are obtained by dividing the corresponding product by the aperture electrical area in square wavelengths ($\lambda _L^2$) at the lowest frequency of the operating band. In fact, GBP/A is exactly $(4.3\pi /100)$ times the product of aperture efficiency and percentage bandwidth. Table 3 indicates that the RCA with DF-PTS has the highest GBP, GBP/A, and DBP/A. They are 1228, 38.93 and 45.74, respectively. It also has the largest measured 3-dB gain and directivity bandwidths of 12.0%. The measured DBP, DBP/A, GBP and GBP/A of the demonstrated system are 68%, 16%, 99% and 37% greater, respectively than those of the most closely related dielectric-free structure based antenna reported in^38^.

**Table 3:** Comparison of measured performance with recently reported PCS/metasurface (MS) based RCA designs.

Ref.	Base antenna	PCS/MS types	Peak directivity (dB)	Peak gain (dB)	3-dB directivity bandwidth (%)	3-dB gain bandwidth (%)	PCS/MS weight	Total		DBP	DBP/A	GBP	GBP/A
								Antenna height ($\lambda _0$)	Electrical area ($\lambda _L^2$)				
**This work**	**RCA**	**Metal**	**20.8**	**20.1**	**12.0**	**12.0**	**150**	**1.50**	**31.54**	**1443**	**45.74**	**1228**	**38.93**
^17^	RCA	Printed	20.2	19.3	6.4	–	190	1.06	32.76	670	20.48	–	–
^31^	RCA	Dielectric	21.6	20.6	8.0	$\sim$8.0	335	2.30	32.76	1156	35.29	919	28.03
^32^	RCA	Printed	21.0	$\sim$20.8	11.8	$\sim$9.4	193	1.01	32.76	1486	45.34	1130	34.49
^33^	RCA	Dielectric	22.0	–	–	–	250	1.74	–	–	–	–	–
^36^	RCA	Printed	21.49	20.78	–	$\sim$3.4	–	1.00	11.46	–	–	407	35.50
^38^	RCA	Metal	$\sim$19.8	19.4	$\sim$9.0	$\sim$7.1	87	1.65	21.80	859	39.35	618	28.31

‘–’ values are not available in the literature.

‘$\sim$’ estimated from the wideband graphs in the literature.

## Conclusion

A mechanically robust and wideband dielectric-free phase transforming structure (DF-PTS) is presented for near-field phase correction of a resonant-cavity antenna, and its wideband performance is successfully demonstrated by testing a prototyped DF-PTS and RCA. The final antenna system has a measured 3-dB gain bandwidth of 12.0%, which unprecedented for printed multilayered phase correction. It represents a 1.7-fold increase in percentage bandwidth over the previous dielectric-free phase correction structure and 1.27-fold increase over the most wideband printed multilayered phase correction structure. The reason for this outstanding bandwidth enhancement is due to two unique characteristics of the new DF-PTS: (i) Each DF-PSC in significant areas of the RCA aperture (i.e. 5 out of 6 cells) has a phase response that tracks reasonably well the phase error variation of the RCA with frequency over a large bandwidth; (ii) Transmission through each of the said cells is very good over the same bandwidth. When compared with previous methods applied for phase correction of RCAs, the demonstrated system has the highest Gain-Bandwidth Product per unit Area (GBP/A) and the highest Aperture-Efficiency-Gain-Bandwidth product, indicating more efficient utilization of antenna area. The lack of dielectrics not only reduced the cost and weight of the DF-PTS but also made it suitable for future space-borne systems where dielectric ionization is critical and high-power systems where a dielectric breakdown is possible. The overall highest gain bandwidth, GBP, GBP/A, and DBP/A along with the aforementioned superior attributes, make the new DF-PTS suitable for many emerging high-gain wideband applications.
